# Semi-automatic inline calculation of left ventricular function using cardiac MRI (CMR)

**DOI:** 10.1186/1532-429X-15-S1-P80

**Published:** 2013-01-30

**Authors:** Audrey M Sigmund, Asad Usman, Marie Wasielewski, Arvin R Akhavan, Shivraman Giri, Matthew A Stratton, Jeremy Collins, James Carr

**Affiliations:** 1 Radiology, Northwestern University, Chicago, IL, USA; 2 Siemens Healthcare, Chicago, IL, USA

## Background

Cardiac Magnetic Resonance (CMR) imaging is increasingly recognized as the gold standard for evaluating left ventricular function (LVF). Measurement of LVF is typically done manually by tracing endocardial and epicardial borders on a stack of short axis (SA) cine images of the heart and is based on the modified Simpson assumption that the left ventricle approximates a spherical shape. A drawback of this method is that it is laborious and time-consuming. The recent introduction of computer-based inline VF software that allows for automatic assessment raised the prospect for significant reductions in CMR post processing times. Initial results have found that fully automatic analysis is unreliable as compared to manual calculations. However, usually only 1 or 2 contours are inaccurate using the automatic software suggesting that, if these are corrected manually, a semi-automatic approach may be helpful. This study sought to compare fully automatic inline VF tracking to this semi-automatic method using the manual technique as gold standard.

## Methods

This was a retrospective cohort study of subjects scanned on a 1.5 T MRI scanner (MAGNETOM Aera Siemens, Germany) from January 1, 2012 to July 7, 2012. 8-12 SA steady state free precession slices from the base to apex of the heart were acquired for each subject and automatic inline calculations of LVF were performed. 77% of patients received gadolinium contrast prior to SA cine acquisition to shorten overall procedure time.

**Figure 1:**
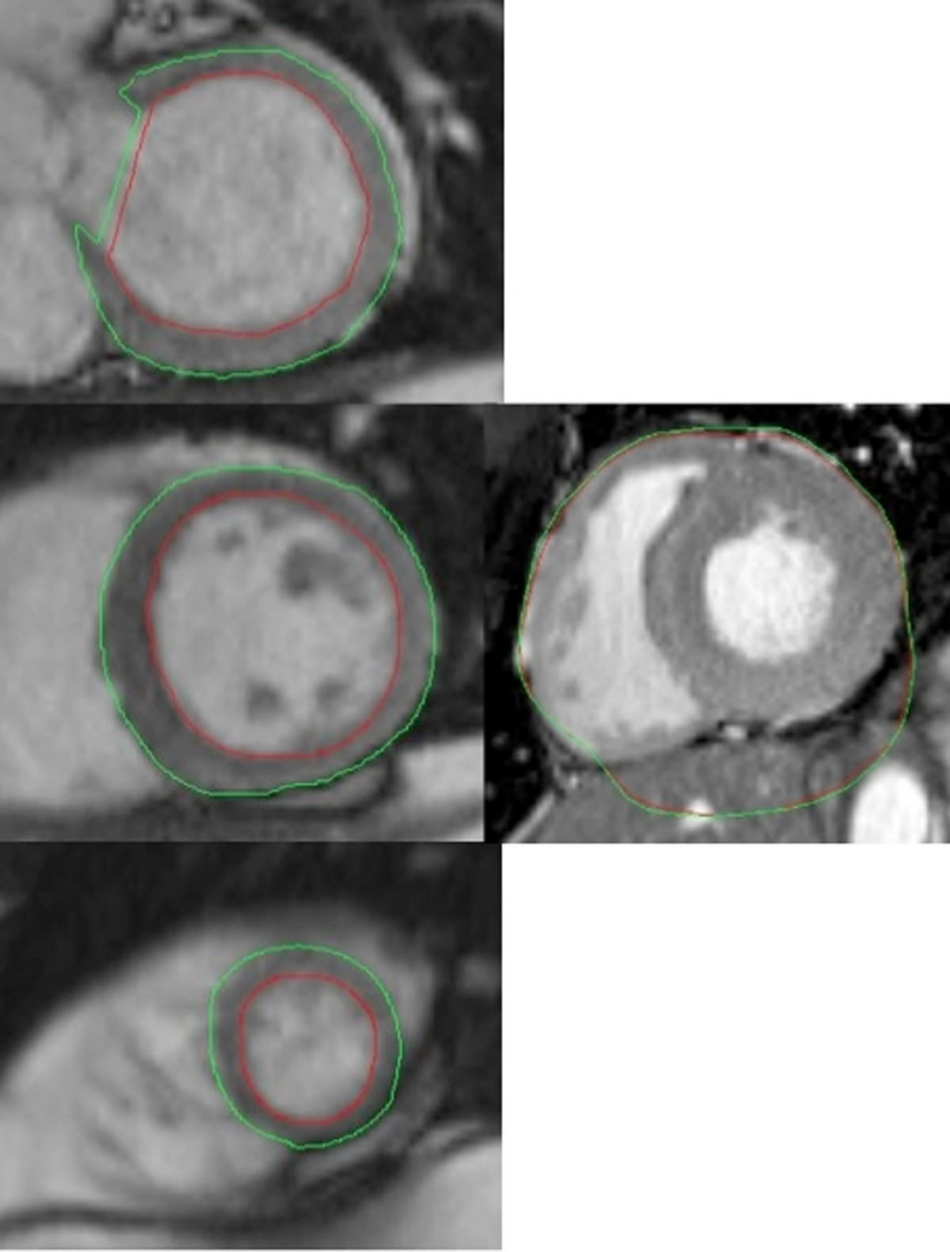
Left: Semiautomatic contours drawn from basal (top) to apical (bottom) slices. Right: Image showing a case where the automated inline tracking failed, misidentifying the whole heart as the left ventricle.

The SA cine images with automatic contours were then transferred to an independent workstation (Leonardo, Siemens Medical Solutions) and the contours were corrected using standard software (Argus, Siemens), if needed, by 2 independent observers (AS, MW). This constituted the semi-automatic analysis (Figure 1). Original SA cine images, without contours, were also analyzed manually by 2 independent observers (AS, MW) in the usual manner with Argus software. LVF parameters and measuring times were recorded for the three techniques and inter-observer and intra-observer correlation was calculated.

## Results

100 subjects were included, with 38% female and 62% male and a mean age of 54.13 ± 14.94. Fully automated inline tracking failed for 9 cases. For the 91 successful cases, an overview of the results is presented in Table 1. Mean EF for automatic, semi-automatic, and manual were 50.6%, 60.2%, and 61.8%, respectively, and mean ESV was 79.6 mL, 58.30 mL, and 56.9 mL. Mean measuring times for semiautomatic and manual techniques differed significantly, averaging 2:20 and 5:11, respectively.

**Table 1:** 

Parameters	Automatic	Semi-automatic	Manual
EF (%)	50.60 ± 10.46	60.20 ± 9.83	61.80 ± 9.53
EDV (mL)	157.30 ± 55.89	140.60 ± 49.70	143.30 ± 51.01
ESV (mL)	79.60 ± 40.74	58.30 ± 33.10	57.0 ± 32.54
SV (mL)	77.80 ± 28.22	82.20 ± 25.44	86.30 ± 27.64
CO (L/min)	4.90 ± 1.68	5.20 ± 1.57	5.50 ± 1.69
Mean Measuring Time (min:sec)	0	2:20	5:11

## Conclusions

There was consistent underestimation of EF with automatic LVF analysis compared to semi-automatic and manual approaches, but semi-automatic and manual measurements were strongly correlated. There was significant reduction in post processing time with semi-automatic as compared to manual analysis. Semi-automatic analysis of LVF is accurate compared to the manual method and may improve workflow by reducing post processing time.

## Funding

Not funded.

